# The Antifungal Activity of HMA, an Amiloride Analog and Inhibitor of Na^+^/H^+^ Exchangers

**DOI:** 10.3389/fmicb.2021.673035

**Published:** 2021-05-05

**Authors:** Kiem Vu, Eduardo Blumwald, Angie Gelli

**Affiliations:** ^1^Department of Pharmacology, School of Medicine, Genome and Biomedical Sciences Facility, University of California, Davis, Davis, CA, United States; ^2^Department of Plant Sciences, University of California, Davis, Davis, CA, United States

**Keywords:** *Cryptococcus*, antifungal, amiloride, HMA, synergy, Nhx1, azoles, Na^+^/H^+^ exchanger

## Abstract

One path toward identifying effective and easily accessible antifungals is to repurpose commonly used drugs. Amiloride, a widely used diuretic, inhibits different isoforms of Na^+^/H^+^ exchangers, Na^+^ channels, and Na^+^/Ca^2+^ exchangers. Here, we found that amiloride had poor antifungal activity against isolates of *Cryptococcus* prompting the examination of the amiloride analog, HMA [5-(*N*,*N*-hexamethylene)amiloride]. HMA possesses strong activity against Na^+^/H^+^ exchangers (NHEs) and little K^+^-associated toxicity since HMA has only minimal inhibitory effects toward epithelial sodium channels (ENaC), the diuretic and antikaliuretic target of amiloride. Although HMA produced a robust dose-dependent growth inhibition of several fungal isolates, susceptibility assays revealed modest MICs against isolates of *Cryptococcus*. A checkerboard dilution strategy resulted in fractional inhibitory concentrations (FIC) < 0.5, suggesting that HMA displays synergy with several antifungal azole drugs including posaconazole, voriconazole, and ketoconazole. Itraconazole and ravuconazole showed moderate synergy with HMA across all tested fungal isolates. In combination with HMA, ravuconazole had MICs of 0.004–0.008 μg/ml, a ∼16-fold reduction compared to MICs of ravuconazole when used alone and significantly more effective than the overall MIC_90_ (0.25 μg/ml) reported for ravuconazole against 541 clinical isolates of *Cryptococcus neoformans*. In combination with azole drugs, MICs of HMA ranged from 3.2 μM (1 μg/ml) to 26 μM (16 μg/ml), HMA was not cytotoxic at concentrations ≤ 8 μg/ml, but MICs were above the reported HMA K_i_ of 0.013–2.4 μM for various Na^+^/H^+^ exchangers. Our results suggest that HMA has limited potential as a monotherapy and may have additional targets in fungal/yeast cells since strains lacking NHEs remained sensitive to HMA. We determined that the hydrophobic substituent at the 5-amino group of HMA is likely responsible for the observed antifungal activity and synergy with several azoles since derivatives with bulky polar substitutions showed no activity against *Cryptococcus*, indicating that other 5-substituted HMA derivatives could possess stronger antifungal activity. Moreover, substitution of other positions around the pyrazine core of HMA has not been investigated but could reveal new leads for antifungal drug development.

## Introduction

Invasive fungal infections continue to be a serious threat to human health having claimed 1.6 million deaths annually in recent years (Almeida et al., 2019). Individuals suffering from severe fungal disease have topped one billion but a lack of compulsory surveillance suggests that cases are likely underreported (Almeida et al., 2019). The *Cryptococcus* spp. complex includes at least seven distinct species that can cause life-threatening disease in immunocompromised and immunocompetent individuals (Kwon-Chung et al., 2017). In regions where HIV infection is prevalent, cryptococcal meningitis is the most common form of adult meningitis (Zuger et al., 1986; Rajasingham et al., 2017).

Serious issues with current antifungal drugs are contributing to the challenges associated with resolving life-threatening fungal infections. The repertoire of antifungal drugs is paltry and the increase in resistance is eliminating their usefulness (Smith et al., 2015; Mpoza et al., 2018). Access to all antifungals is limited and in many cases the countries with the most dire need are unable to acquire the most efficacious drugs because of acquisition costs or counterfeit drugs—an increasingly serious threat to resolving fungal disease in resource-poor nations (Loyse et al., 2013; Africa and Abrantes, 2016).

Azoles are undoubtedly the most common antifungal drugs used in clinical practice. Due to their broad spectrum of activity, azoles are used to treat and prevent a number of different mycoses (Campoy and Adrio, 2017). The antifungal activity of azoles is attributed to their ability to inhibit 14α-lanosterol demethylase, a cytochrome P450-dependent enzyme (CYP51) (Campoy and Adrio, 2017). Encoded by the *ERG11* gene, lanosterol demethylase changes lanosterol to ergosterol, but in the presence of azoles ergosterol is depleted from the cell membrane resulting in inhibition of fungal growth and replication (Odds et al., 2003; Campoy and Adrio, 2017). Several new azoles have emerged recently, but fluconazole which has been commercially available since 1988, is by far the most common antifungal used in resource-poor countries due to its efficacy and low acquisition costs (Loyse et al., 2013). However, its efficacy as a monotherapy for cryptococcal meningitis is limited and the development of fluconazole resistance in *Candida* spp. is further eroding its usefulness (Molloy et al., 2018). A major drawback of fluconazole, like some other azoles, is its mere fungistatic activity, which has prompted a search for other drugs/compounds that can be used in combination with azoles to produce desirable fungicidal effects (Marchetti et al., 2000; Onyewu et al., 2003; Vu and Gelli, 2010; Butts et al., 2017).

One path toward finding effective and easily accessible antifungals is to repurpose commonly used drugs (Kim et al., 2020). Amiloride is a pyrazine compound with a guanidinium-substituent and was initially discovered as an inhibitor of Na^+^/H^+^ exchangers (NHEs) (Benos, 1982). NHEs represent a large family of integral membrane proteins that contribute to the acidification of the lumen of intracellular organelles (Nakamura et al., 2005). It is widely known that amiloride inhibits different isoforms of NHEs with an IC_50_ of 5.3–50 μM and also inhibits epithelial Na^+^ channels and Na^+^/Ca^2+^ exchangers (Cragoe et al., 1967; Li et al., 1985; Palandoken et al., 2005). This lack of specificity prompted the synthesis of several analogs of amiloride by a double substitution on the 5-amino group resulting in HMA [5-(*N*,*N*-hexamethylene)amiloride], EIPA [5-(*N*-ethyl-*N*-isopropyl)amiloride], and MIA [5-(*N*-methyl-*N*-isobutyl)amiloride] (Masereel et al., 2003). Among the analogs, HMA [5-(*N*,*N*-hexamethylene)amiloride], is the most active since it specifically inhibits NHEs, and has only minimal inhibitory effects against the epithelial sodium channels (ENaC), the diuretic and antikaliuretic target of amiloride (Cragoe et al., 1967) (Masereel et al., 2003).

From a clinical perspective, amiloride is used as a potassium sparing drug because it blocks Na^+^ re-absorption by inhibiting epithelial Na^+^ channels (ENaCs) in the distal tubules of the nephron of kidneys with an IC_50_ of ∼0.1–0.5 μM (Kleyman and Cragoe, 1988). Normally, sodium reabsorption *via* ENaCs promotes excretion/loss of K^+^, but excessive Na^+^ reabsorption increases loss of K^+^, resulting in hypokalemia. The potent inhibition of ENaCs by amiloride supports its use as a diuretic because it prevents K^+^ loss/excretion and decreases water retention, however, this K^+^-sparing effect can lead to hyperkalemia (Vidt, 1981).

Our goal was to examine whether amiloride could be repurposed for the treatment of fungal infections given that amiloride is widely used in clinical practice, can penetrate the blood-brain barrier, is well-tolerated and accessible. Our impetus for this study was based on the pressing need to develop novel antifungal drugs that can enter the central nervous system, are efficacious and safe. In this study amiloride and its analogs were examined for antifungal activity and their potential use in combination therapy with existing azoles was assessed using a checkerboard dilution strategy to determine fractional inhibitory concentrations (FICs), an *in vitro* measure of drug synergy.

## Results

**FIGURE 1:**
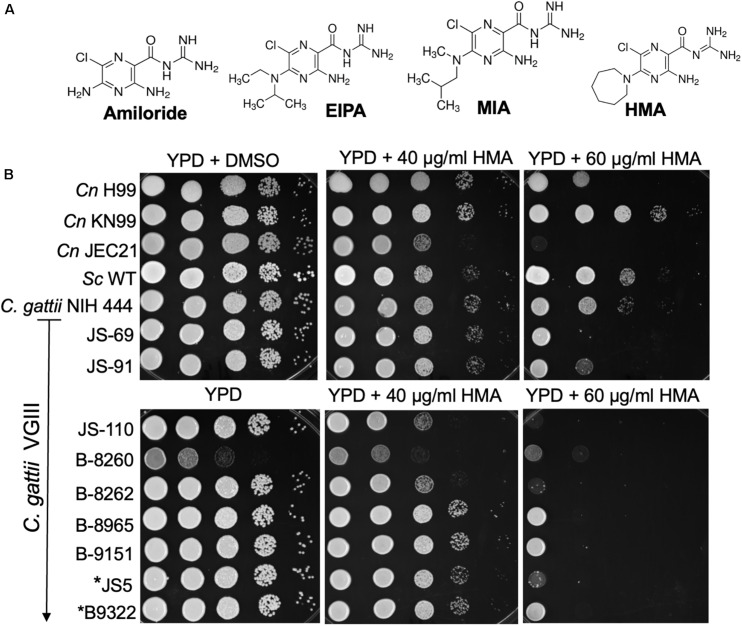
Cryptococcus demonstrates a dose-dependent growth inhibition to HMA. **(A)** Amiloride and three analogs: EIPA: [5-(*N*-ethyl-*N*-isopropyl) amiloride]; MIA: [5-(*N*-methyl-*N*-isobutyl)amiloride]; HMA: [5-(*N*,*N*-hexamethylene) amiloride]; **(B)** *C. neoformans* var. *grubii* (H99 and KN99), *C. neoformans* var. *neoformans* (JEC 21, serotype D), and ten *Cryptococcus gattii* clinical isolates (ATCC NIH444, JS-69, JS-91, JS-110, B-8260, B-8262, B-8965, B-9151, JS5, and B9322, kindly provided by G. R. Thompson) were examined for their susceptibility to HMA in spot sensitivity assays,. *JS5 and *B9322 were previously identified as flucytosine resistant clinical isolates (Vu et al., 2018). *Sc* – *Saccharomyces cerevisiae*, W303a lab strain. Image from three replicates.

Initially we examined whether amiloride possessed antifungal activity. Susceptibility assays revealed an MIC > 278 μM (>64 μg/ml) for amiloride against *Cryptococcus neoformans* (Table 1). The lack of significant antifungal activity of amiloride led us to examine three analogs that were derived by double substitution of the 5-amino group of amiloride and were known to possess greater specificity for NHEs in plants and animals (Cragoe et al., 1967). We tested MIA [5-(*N*-methyl-*N*-isobutyl)amiloride], EIPA [5-(*N*-ethyl-*N*-isopropyl)amiloride] and HMA [5-(*N*,*N*-hexamethylene)amiloride]. Analog of amiloride, MIA [5-(*N*-methyl-*N*-isobutyl)amiloride], EIPA [5-(*N*-ethyl-*N*-isopropyl)amiloride] and HMA [5-(*N*,*N*-hexamethylene)amiloride], showed a better antifungal activity against cultures of *Cryptococcus* spp. than amiloride alone, and HMA resulted the best active compound. Based on these results we examined HMA activity further against several fungal isolates *via* spot sensitivity assays (Figure 1). Of the fungal isolates tested we found that *Cryptococcus neoformans* (*Cn*) and *Cryptococcus gattii* (*Cg*) displayed growth sensitivity in a dose-dependent manner at concentrations of HMA ≥ 40 μg/ml and were no longer viable at concentrations of 60 μg/ml (Figure 1). Interestingly, the clinical isolates of *Cg*, specifically JS-110, B-8260, B-8262, and JS5, displayed a more severe growth defect at 60 μg/ml compared to *Cn* KN99 and *Cn* H99 isolates (Figure 1). The *Cg* JS5 and B9322 isolates that were resistant to flucytosine, an antifungal drug, also displayed a severe growth sensitivity to HMA at 60 μg/ml.

**TABLE 1:**
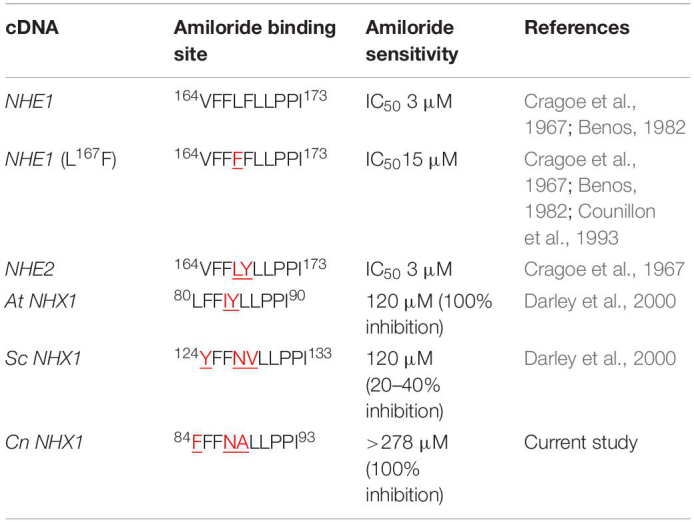
Comparison of the amiloride binding site in Na^+^/H^+^ exchangers of *Cryptococcus neoformans*, yeast, plants and mammals.

Next we assessed the activity of HMA by microbroth dilution susceptibility assays to determine the minimum inhibitory concentration (MIC) of HMA. We found that HMA exhibited only minimal fungicidal activity, albeit stronger than amiloride, against three common *Cryptococcus* isolates (Table 2). The MIC values for HMA against *Cn* H99, *Cn* JEC21, and *Cg* NIH444 were between 32 and 64 μg/ml (Table 2). Since the relatively high MICs of HMA preclude its use as a monotherapy, we examined the possibility of a combination therapy with current azoles. To assess for the presence of synergy between HMA and the azole class of antifungals, the FIC, was used as a measure of drug synergy (Johnson et al., 2004). Several FIC indices were <0.5, suggesting that HMA exhibited moderate synergistic activity with itraconazole, ketoconazole, posaconazole, ravuconazole, and voriconazole (Table 2).

**TABLE 2:** Synergy activity of HMA with azole antifungal drugs against strains of *Cryptococcus: Cryptococcus neoformans* H99, *Cryptococcus neoformans* JEC21, and *Cryptococcus gattii.*

	***Cryptococcus neoformans* H99**			***Cryptococcus neoformans* JEC21**			***Cryptococcus gattii* NIH444**		
**Drug**	**MIC (i)**	**MIC (t)**	**FIC**	**MIC (i)**	**MIC (t)**	**FIC**	**MIC (i)**	**MIC (t)**	**FIC**
HMA	64	16	0.50	32	8	0.50	64	8	0.63
Flue	4	1		2	0.5		2	1	
HMA	64	1	0.27	32	4	0.38	32	2	0.13
Itra	0.25	0.063		0.25	0.063		4	0.25	
HMA	32	8	0.50	64	16	0.37	64	16	0.38
Keto	0.125	0.031		0.125	0.016		0.5	0.063	
HMA	32	4	0.25	64	16	0.28	64	32	0.62
Posa	0.125	0.016		0.25	0.008		0.125	0.016	
HMA	32	2	0.13	32	4	0.19	32	8	0.31
Ravu	0.063	0.004		0.125	0.008		0.125	0.008	
HMA	32	2	0.31	32	8	0.50	64	8	0.37
Vori	0.063	0.016		0.031	0.008		0.063	0.016	

*MIC(t) MIC (μg/ml) of drugs together; MIC(i), MIC (μg/ml) of individual drug; FIC, fractional inhibitory concentration. Drug interactions were based on ΣFIC indices and classified as synergistic (Σ FIC < 0.5), additive (Σ FIC = 0.5 through 1), indifferent (Σ FIC = 1 through 4), or antagonistic (Σ FIC > 4). Fluc, fluconazole; Itra, itraconazole; Keto, ketoconazole; Posa, posaconazole; Ravu, ravueonazole; Vori, voriconazole. HMA, 5-(*N*,*N*-Hexamethylene)amiloride. Susceptibility assays were repeated atleast three times.*

Two azole drugs displayed stronger synergistic activity with HMA (FIC index = 0.13, Table 2). The FIC index for HMA plus ravuconazole or itraconazole was 0.13 at 35°C for *Cn* H99 and *Cg*, respectively. To illustrate the extent of synergy, the MICs of HMA and ravuconazole given alone were 32 and 0.063 μg/ml, respectively, whereas in combination, the MICs of HMA and ravuconazole decreased to 2 and 0.004 μg/ml, respectively (Table 2). Therefore, in combination, the effective dose of HMA and ravuconazole was ∼16-fold lower. In combination with HMA the MICs of itraconazole, ketoconazole, voriconazole, and posaconazole were reduced between 4- and 32-fold against isolates of *Cg* and/or *Cn* (Table 2). The MIC of HMA was reduced between 64-fold and 4- when combined with various azole drugs against *Cg* and/or *Cn* (Table 2).

**FIGURE 2:**
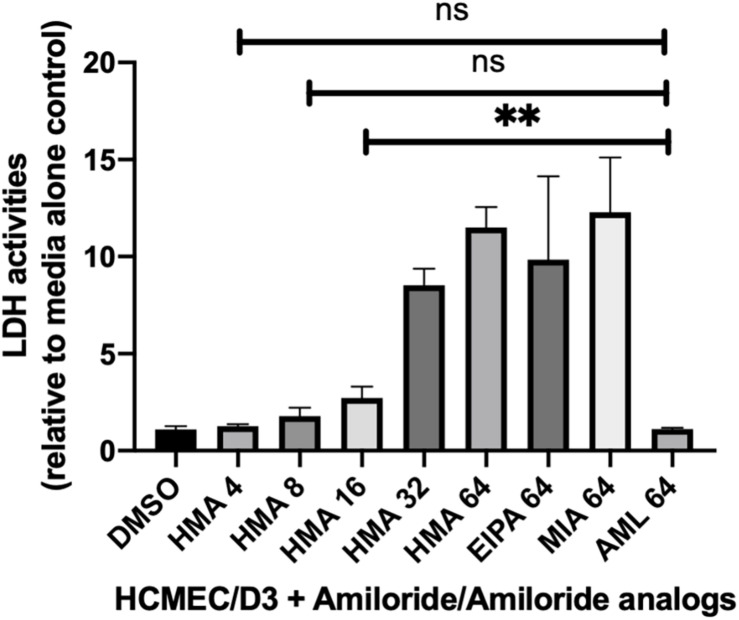
Lactate dehydrogenase (LDH) activity assays in mammalian cells treated with HMA indicate little to no cytotoxicity at concentrations of HMA ≤ 8 μg/ml. The human cerebral microvascular endothelial cell line (HCMEC/D3) was treated with either amiloride (AML) or the amiloride analogs (HMA, EIPA, MIA) at the indicated concentrations (4, 8, 16, 32, or 64 μg/ml) for 8 h prior to measuring LDH activity. The cytotoxicity of HMA at 8 or 4 μg/ml is not significantly different from that of amiloride at 64 μg/ml (*P* > 0.05). Data shown is from three replicates ± SD; ns, not significant.

The synergy of HMA with several azole drugs, led us to assess the clinical potential of HMA as an antifungal therapeutic by examining its cytotoxicity profile (Figure 2). HMA was significantly more cytotoxic than amiloride at concentrations ≥ 16 μg/ml (*P* < 0.01) (Figure 2); however, cytotoxicity was greatly reduced at concentrations of HMA that were synergistic with azoles and not significantly different from that of amiloride (*P* > 0.05).

**FIGURE 3:**
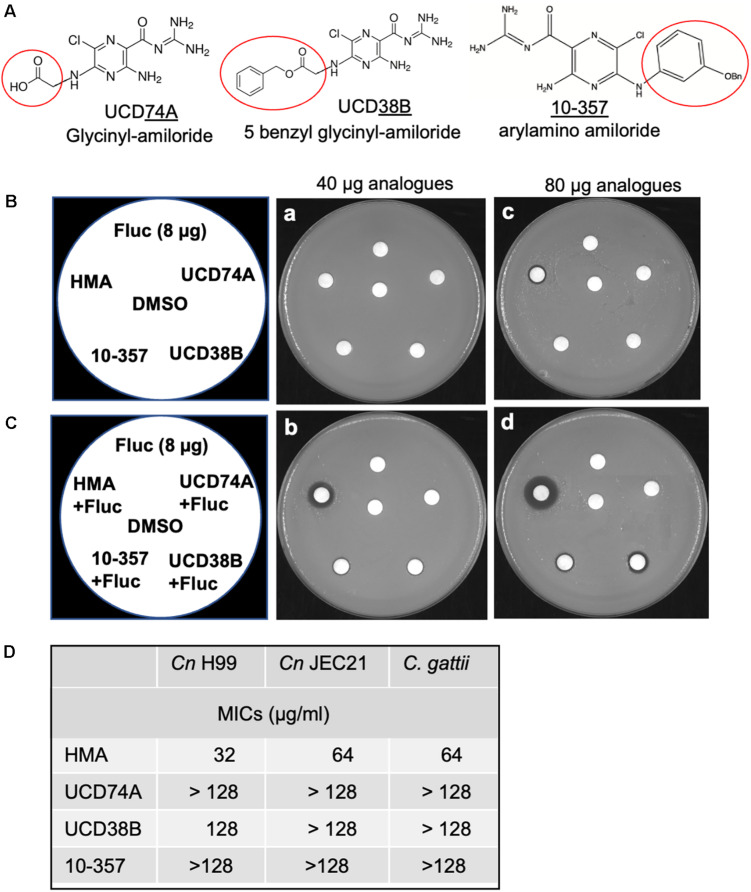
Polar substitutions at the 5-amino group of amiloride eliminate antifungal activity. **(A)** Three amiloride analogs UCD38B (5-benzyl glycinyl-amiloride), UCD74A (glycinyl-amiloride), and 10-357 (arylamino amiloride, Bn, benzyl group) with polar group substitutions at the 5-amino group of the pyrazine ring (kindly provided by F. Gorin, UC Davis). **(B,C)** Disk diffusion assays showing susceptibility of the *C. neoformans* H99 strain to HMA and three other amiloride analogs at 40 and 80 μg and fluconazole (Fluc) at 8 μg either alone **(B)** or in combination with fluconazole (8 μg) **(C)**. **(D)** Susceptibility of *Cryptococcus neoformans* H99, *Cryptococcus neoformans* JEC21 serotype D, and *Cryptococcus gattii*, to amiloride analogs HMA, UCD74A, UCD38B, and 10-357 as determined by MICs. Susceptibility assays were performed at least three times.

To address whether HMA-like analogs might possess more potent antifungal and/or synergistic activity, we examined additional analogs with R-group substitutions at the 5-amino group (Figure 3A). We examined three analogs with bulky polar substitutions at the 5-amino group by disk diffusion assays (Figure 3). We found that analogs UCD74A, UCD38B, and 10-357 (glycinyl-amiloride, five benzyl glycinyl-amiloride, and arylamino amiloride, respectively) had no antifungal activity on their own (Figures 3B,D) and little to no activity in combination with azole drugs based on the absence of a halo for UCD74A and the very minimal halo observed for 10-357 and UCD38B (Figure 3C). We identified several additional analogs *in silico* with extensive hydrophobic substitutions at the 5-amino group (Supplementary Figure 1) that we intended to examine for antifungal activity, however, these compounds were unavailable (Supplementary Figure 1).

**FIGURE 4:**
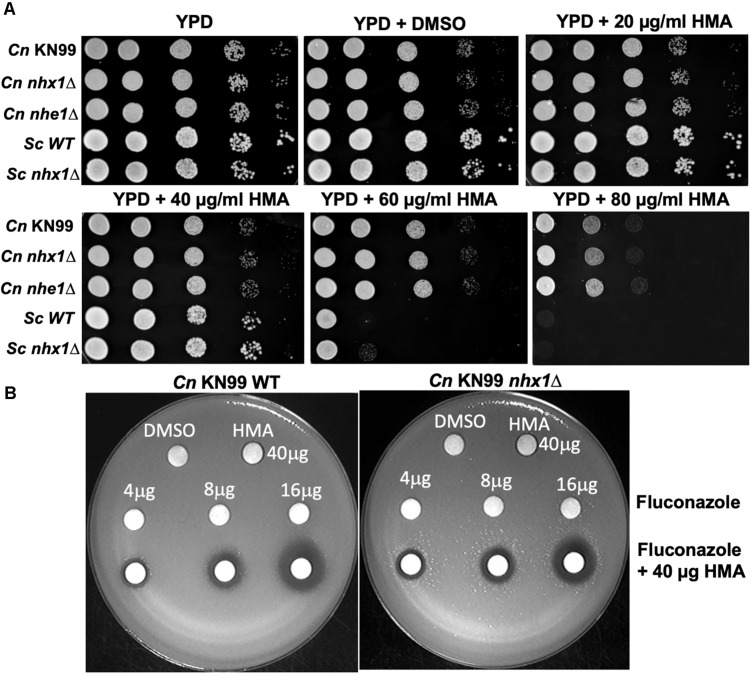
Fungal/yeast cells lacking Na^+^/H^+^ exchangers, Nhx1 or Nhe1, are sensitive to HMA. **(A,B)** Growth sensitivity assays of the *C. neoformans* KN99 wild type strain, *nhx1Δ* and *nhe1Δ* strains demonstrate a similar dose-dependent response to HMA. The background strain of *nhx1Δ* and *nhe1Δ* is KN99. *Sc* – *Saccharomyces cerevisiae* W303 **a** strain. Background strain of *Sc nhx1Δ* is W303 a. **(B)** Disk diffusion assay shows a similar response of KN99 wild type and *nhx1Δ* strains to HMA with or without fluconazole. A lawn of KN99 wild type and *nhx1Δ* strains were tested with either HMA (40 μg), fluconazole (4–16 μg) or fluconazole (4–16 μg) in combination with HMA (40 μg HMA). Images shown are representative of at least three replicates.

**FIGURE 5:**
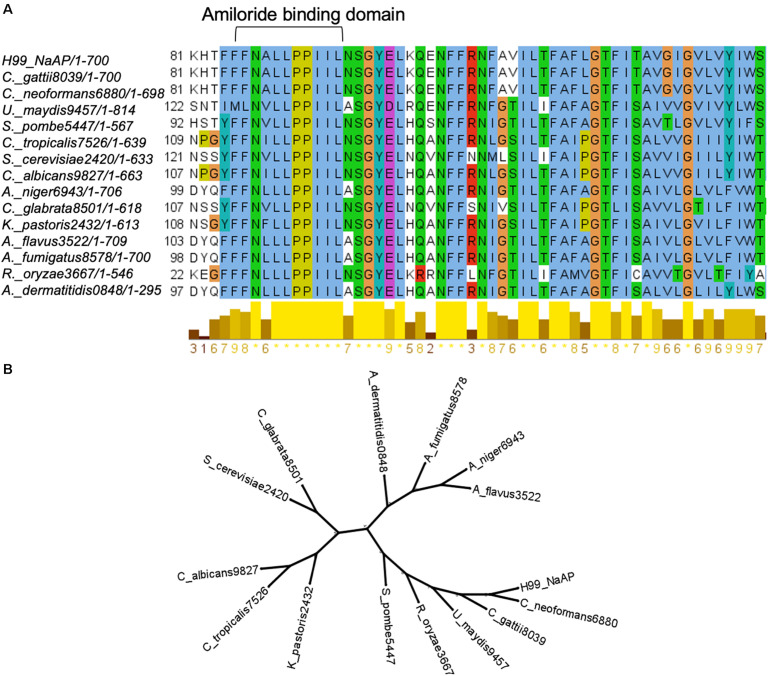
The intracellular Na^+^/H^+^ exchanger, Nhx1, in fungi. **(A)** Amiloride binding domain in Nhx1 is highly conserved among fungi. **(B)** Phylogenetic analysis of Nhx1 protein sequences from 13 fungal species and *S. cerevisiae* indicates the relative conservation of Nhx1 among these species.

Since HMA is a well-known specific inhibitor of NHEs in mammalian cells, we aimed to determine whether *Cn* Nhx1, the NHE endosomal exchanger in fungi/yeast, was a direct target of HMA. We reasoned that if HMA inhibited Nhx1 activity directly, then there should be little to no effect of HMA on the growth of *nhx1Δ* deletion strains. To test this, the growth of both *Sc nhx1*Δ and *Cn nhx1Δ* strains were examined in the presence of 20–120 μg/ml of HMA (Figure 4). We found that *Sc* and *Cn nhx1Δ* strains had a dose-dependent reduction in growth in the presence of HMA in a manner similar to the response of WT cells (Figure 4A). In addition, disk diffusion assay showed a similar response of *Cn* KN99 and *Cn nhx1Δ* strains to HMA with or without fluconazole (Figure 4B). Given these results, we questioned whether the plasma membrane Na^+^/H^+^ antiporter, *Cn* Nhe1, was a target of HMA since the mammalian plasma membrane Na^+^/H^+^ antiporters, Nhe1 and Nhe2, are known to have the highest sensitivity to amiloride (Figure 4A). Amino acid sequence analysis of *Cn* Nhe1 indicated that *Cn* Nhe1 did not appear to contain any obvious amiloride binding domain (ABD) (data not shown). Consistent with *Cn* Nhe1 lacking a known binding domain for amiloride, the *Cn nhe1Δ* strain had a similar growth defect as WT cells when exposed to 20–120 μg/ml of HMA (Figure 4A). We determined that the putative (ABD) in Nhx1, had significant sequence similarity among fungi with a common origin (Figure 5); however, the ABD in *Sc* Nhx1 and *Cn* Nhx1, which have markedly reduced amiloride sensitivity, differed significantly from that in other NHEs (Table 1; Counillon et al., 1993).

## Discussion

The aim of this study was to determine whether amiloride, a commonly used diuretic, could be repurposed as an antifungal drug. Our studies found that amiloride had little to no antifungal activity at concentrations that would be practical since its activity as a diuretic would supersede any antifungal activity. We questioned whether the noted specificity of HMA, an amiloride analog, for NHEs would promote or improve its antifungal activity and decrease any K^+^-associated toxicities that might lead to hyperkalemia since HMA is only minimally active toward ENaC. Our results demonstrated that HMA had weak fungicidal activity, although greater than amiloride, as indicated by the MIC values against several isolates of *C. neoformans* and *Cryptococcus gattii*. This result along with the LDH activity observed in mammalian cells treated with similar concentrations of HMA and the apparent K_i_ of HMA for Na^+^/H^+^ exchangers would not support the use of HMA as a monotherapy for cryptococcal infections.

We did find, however, that HMA possessed moderate synergy with posaconazole, voriconazole and ketoconazole, although the synergy was strain dependent. Posaconazole and voriconazole appear to be more potent than either itraconazole or fluconazole against published clinical isolates of *C. neoformans* from Africa and the United States, however, posaconazole has limited penetration of the CNS (Pfaller et al., 1999, 2004). HMA displayed synergistic activity with ravuconazole and itraconazole against all strains tested and our reported MICs were significantly more effective than the overall MIC_90_ (0.25 μg/ml) reported for ravuconazole against 541 clinical isolates of *C. neoformans* (Yamazumi et al., 2000). We found that HMA was not cytotoxic at concentrations that promote synergy with the azole antifungals, however, these MICs were above the reported K_i_ of 0.013–2.4 μM for HMA against various Na^+^/H^+^ exchangers (Kleyman and Cragoe, 1988).

HMA belongs to the amiloride class of pyrazines, in which the two hydrogens at the 5-amino position (adjacent to Cl^–^) are substituted with a hexamethylene moiety resulting in the formation of an azepane ring (Cragoe et al., 1967). Early work by Cragoe et al. (1967) found that substitutions of the H-atoms at the 5-amino group with hydrophobic R-groups resulted in 3–200-fold increase in the inhibitory potency of the Na^+^/H^+^ exchanger activity (Blumwald et al., 1987; Kleyman and Cragoe, 1988). Consistent with this result, we found that polar substitutions of the 5-amino group (i.e., 5-benzyl glycinyl-amiloride, glycinyl-amiloride, or arylamino amiloride) had no antifungal activity indicating that a hydrophobic substituent at the 5-amino group, such as the 5-hexamethylene group in HMA, was likely responsible for the observed antifungal activity. However, we observed that strains lacking Nhx1 or Nhe1, remained sensitive to HMA, suggesting that the Na^+^/H^+^ exchangers may not be targets of HMA in fungal/yeast cells, despite the increased potency of HMA for NHEs in higher eukaryotes.

The lack of sequence similarity we noted in the binding domain of amiloride/HMA in NHEs across mammals, plants, and fungi/yeast would further support the presence of other HMA targets in fungi. Mutational analysis of the amino acid at position 4 and 5 of the ABD suggested that a leucine (L) residue at position 4 and either a phenylalanine (F) or tyrosine (Y) residue at position 5 were important for amiloride inhibition (Counillon et al., 1993). In the case of *Cn* Nhx1, differences in the amino acids at these key positions may have precluded the binding of amioride/HMA. Nevertheless, we could not rule out the possibility that HMA may have indeed inhibited *Cn* Nhx1 or *Cn* Nhe1, but other cation/proton exchangers may have compensated (Cagnac et al., 2007). Cagnac et al. (2007) identified a novel type of vacuolar monovalent cation-proton exchanger, Vnx1p, in *Saccharomyces cerevisiae* with low affinity to Na^+^ and K^+^ despite its homology to the CAX (calcium/proton exchangers) family. Similar to Nhx1, the Vnx1 exchanger could be associated with the regulation of ion homeostasis and cellular pH in yeast (Cagnac et al., 2007). A search for *VNX1* in the *Cryptococcus* genome identified an uncharacterized open reading frame listed as a calcium/proton exchanger with 32% amino acid identity to *Sc* Vnx1. This could be indicative of a Vnx1-like protein in *Cn*, supporting the notion of a compensatory mechanism active in the absence of Nhx1 activity.

Alternatively, off-target effects of HMA in fungal cells could be possible and would be consistent with other reports that demonstrated HMA activity toward G-protein-coupled receptors, such as the adenosine 2A receptor (Garritsen et al., 1991; Gao and Ijzerman, 2000; Soudijn et al., 2004). The reported anticancer activity of HMA and its analogs stems from their antitumor/metastasis effects due in part to the inhibition of the human urokinase plasminogen protease that functions as a major driver of cell invasiveness (Kleyman and Cragoe, 1988; Aredia et al., 2016; Buckley et al., 2018). These various biological activities of HMA may be indicative of other targets in fungal cells, possibly in addition to Nhx1 or Nhe1.

The goal of our study was to examine the antifungal activity of amiloride and its analogs, and although we originally thought that Nhx1 was targeting by amiloride/HMA, our results suggest otherwise. It should be noted that we previously tested the *Cn nhx1* deletion strain in media that was supplemented with various concentrations of NaCl, KCl, CaCl_2_ and a range of pH values to amplify any effect of amiloride/HMA. In all cases we found no difference in the growth of the *Cn nhx1* deletion strain compared to wild type, further supporting the notion that Nhx1 may not be a target of HMA in *Cryptococcus*. This result is consistent with published data that has clearly shown that HMA has other targets in mammalian cells. There is a possibility, as discussed above, that perhaps HMA does inhibit *Cn* Nhx1 but other exchangers in *Cn* may be compensating, in which case it would be difficult to observe the effect of HMA on Nhx1. Clearly, resolving the mechanism of HMA in *Cn* will require significantly more work and complex transport studies that are beyond the scope of our study.

In conclusion, our study demonstrated that HMA had minimal antifungal activity and moderate synergy with several antifungal drugs. We propose that further derivation of HMA could lead to compounds with significantly greater antifungal activity. The structure-activity relationship trend we observed suggested that the hydrophobic substitution at the 5-amino group of HMA was likely responsible for the antifungal activity and synergy with azoles, indicating that other similar 5-substituted HMA derivatives could possess stronger activity. Moreover, substitution of other positions around the pyrazine core of HMA has not been investigated but could reveal new leads for antifungal drug development.

## Materials and Methods

### Strains and Media

*Cryptococcus neoformans* H99 and *C. gattii* are clinical isolates and were obtained from ATCC (ATCC 208821 and ATTC 32609 (also known as NIH444), respectively). KN99 is a common *C. neoformans* laboratory strain derived from H99 (Nielsen et al., 2003). The JEC21 strain—a *C. neoformans* var. *neoformans*, serotype D—was a gift from Dr. J. Heitman (Duke University) and is also available at ATCC (ATCC^®^ MYA-565). Other *C. gattii* isolates (JS-69, JS-91, JS-110, B-8260, B-8262, B-8965, B-9151, JS5, and B9322), kindly provided by Dr. G.R. Thompson (UC Davis) included in this study belong to the *C. gattii* VGIII major molecular type and had previously undergone whole genome sequencing as part of a larger population genomics survey (Vu et al., 2018). Unless noted otherwise, the NIH444 *C. gattii* strain is used throughout this study. The *nhx1*Δ and *nhe1*Δ deletion strains were obtained from the available deletion library^1^. The *S. cerevisiae nhx1*Δ deletion mutant and wild-type background strain (W303**a**) was a gift from Dr. E. Blumwald (UC Davis). All strains were recovered from −80°C frozen stocks, grown at 30°C and maintained on yeast peptone dextrose media (YPD) except when tested in susceptibility assays.

### Amiloride/Analogs and Antifungals

MIA, EIPA, HMA, and DMA amiloride analogs [5-(*N*-methyl-*N*-isobutyl)amiloride, 5-(*N*-ethyl-*N*-isopropyl) amiloride, 5-(*N*,*N*-hexamethylene) amiloride, 5-(*N*,*N*-(dimethyl)amiloride, respectively] were purchased from Sigma-Aldrich. Compounds UCD74A, UCD38B, and 10-357 (glycinyl-amiloride, five benzyl glycinyl-amiloride, and arylamino amiloride, respectively) were kindly provided by Dr. F. Gorin (UC Davis). All azole drugs were also purchased from Sigma-Aldrich.

### Spot Sensitivity Assays

DMSO and HMA were added to freshly prepared YPD agar at the indicated concentrations and the plates were allowed to set for 1 h prior to addition of fungal strains. Inoculum from overnight cultures were used to start new cultures for 8–10 h on the day of the experiment. Inoculum size were determined using a hemocytometer (Bright-Line^TM^ Hemacytometer, Sigma-Aldrich). Cultures were washed three times with 1X phosphate-buffered saline (PBS) and resuspended in 1X PBS. Serial 10-fold dilutions were prepared (10^1^ cells/5 μl to 10^5^ cells/5 μl) on 96-well plates, and 5 μl of each dilution was spotted on assay plates using a multichannel pipet. The plates were incubated at 30°C for 48 h after which images were taken.

### Antifungal Drug Activity Testing by CLSI Criteria

Checkerboard titrations were performed in order to assess drug interactions according to the Clinical and Laboratory Standards Institute (CLSI). *In vitro* testing was carried out in RPMI 1640 medium containing L-glutamine, without sodium bicarbonate and buffered to pH 7.0 with MOPS in 96-well plates (96-well cell culture cluster, flat-bottom, Costar). Inoculum of *C. neoformans* (100 μl) was prepared in accordance with the CLSI standard (M27-A3), added to the 96-well plates and incubated for 48 h at 35°C without shaking. Readings were taken by visual inspection of the opacity of the wells. The MIC of drugs alone or in combination was defined as the lowest drug concentration in a well at which 100% reduction in optical density was observed compared to the no-drug control well. FICs and FIC indices (Σ FIC) were determined as previously described (Johnson et al., 2004). The FIC index is defined as the sum of the FICs for each of the drugs and the FICs are defined as the MIC of each drug when used in combination divided by the MIC of the drug when used alone (Johnson et al., 2004). Drug interactions were based on Σ FIC indices and classified as synergistic (Σ FIC < 0.5), additive (Σ FIC = 0.5 through 1), indifferent (Σ FIC = 1 through 4), or antagonistic (Σ FIC > 4) (Johnson et al., 2004). Determination of MICs of amiloride and amiloride analogs were repeated at least three times. The checkerboard dilutions used to calculate FIC were done twice with similar results.

### LDH Assay

Toxicity of HMA was measured *via* lactate dehydrogenase (LDH) activity using the LDH assay kit (Sigma-Aldrich). Briefly, LDH activity, which is a marker of cell viability, was measured following 8 h co- incubation of the human brain microvascular endothelial cell line with 4–64 μg/ml of HMA. Absorbance was measured at 490 nm for 30 min at 37°C, taking the highest time-point reading before absorbance exceeded the maximum tested standard. LDH activity was calculated using an NADH standard set provided by the kit.

### Disk Diffusion Assays

Strains were inoculated into liquid medium and grown overnight at 30°C. Approximately 2 × 10^7^ cells were inoculated into 8 ml of top agar pre-warmed at 42°C and subsequently spread onto YPD plates. All drugs and the solvent control were applied to 6-mm BBL disks (Becton Dickinson). These disks were placed on the solidified top agar surface of the YPD plates as indicated and the strains were grown at 30°C for 48 h.

### Phylogenetic Tree Analysis and Clustal Alignment

Nhx1 protein sequence from *C. neoformans* var. *grubii* (H99 serotype A strain) was used in a BLAST search for homologous proteins in a number of fungal species. The top ‘‘hits’’ were collated and analyzed using the freely available Clustal X 2.1 program^2^. Clustal alignment results were viewed and annotated using Jalview^3^. Phylogenetic trees were obtained by entering Clustal alignment data into FigTree version 1.4.0^4^. Accession numbers are provided in Supplementary Figure 2.

### Statistical Analysis

A standard unpaired *t* test was used for comparison of two groups and a one-way analysis of variance (ANOVA) was used when comparing more than two groups. Statistical significance was established at *P* < 0.05 (^∗∗^*P* < 0.01). Statistical analysis was performed with commercially available software (Prism GraphPad version 8.4.3).

## Data Availability Statement

The raw data supporting the conclusions of this article will be made available by the authors, without undue reservation.

## Author Contributions

KV carried out the experiments. AG and EB developed the idea presented, and supervised the study. AG wrote the manuscript. KV and EB assisted with the edits. All authors analyzed and discussed the results and contributed to the final manuscript.

## Conflict of Interest

The authors declare that the research was conducted in the absence of any commercial or financial relationships that could be construed as a potential conflict of interest.

## Footnotes

1 http://www.fgsc.net/crypto/crypto.htm

2 http://www.clustal.org/clustal2/

3 http://www.jalview.org/

4 http://tree.bio.ed.ac.uk/software/figtree/

## Supplementary Material

Supplementary Figure 1 Candidate amiloride analogs with hydrophobic substitutions at the 5-amino group of the pyrazine ring. **(A)** 3-Amino-*N*-(aminoiminomethyl)-5-azacyclotridec-1-yl-6-chloro-2-pyrazinecarboxamide; **(B)** 3-amino-*N*-(aminoiminomethyl)-6-chloro-5-(3,4-dihydro-2(1H)-isoquinolinyl)-2Pyrazinecarboxamide; **(C)** 3-amino-*N*-(aminoiminomethyl)-6-chloro-5-(2,3-dihydro-1H-indol-1-yl)-2-Pyrazinecarboxamide; **(D)** 3-amino-*N*-(aminoiminomethyl)-6-chloro-5-(methylphenylamino)-2-Pyrazinecarboxamide; **(E)** 3-amino-*N*-(aminoiminomethyl)-6-chloro-5-(cyclohexylmethylamino)-2-Pyrazinecarboxamide; **(F)** 3-amino-*N*-(aminoiminomethyl)-6-chloro-5-[(cyclohexylmethyl)amino]-2-Pyrazinecarboxamide; **(G)** 3-amino-*N*-(aminoiminomethyl)-6-chloro-5-(dipropylamino)-2- Pyrazinecarboxamide.

Supplementary Figure 2 Accession numbers for fungal amino acid sequences of Nhx1. Shown are the corresponding accession numbers for Nhx1 for each species of fungi used in the Clustal alignment.
