# Supplementary figures and images for: The Antifungal Activity of HMA, an Amiloride Analog and Inhibitor of Na+/H+ Exchangers
Source: Front Microbiol. 2021 May 5;12:673035. doi: 10.3389/fmicb.2021.673035 (PMC8133316; doi:10.3389/fmicb.2021.673035)

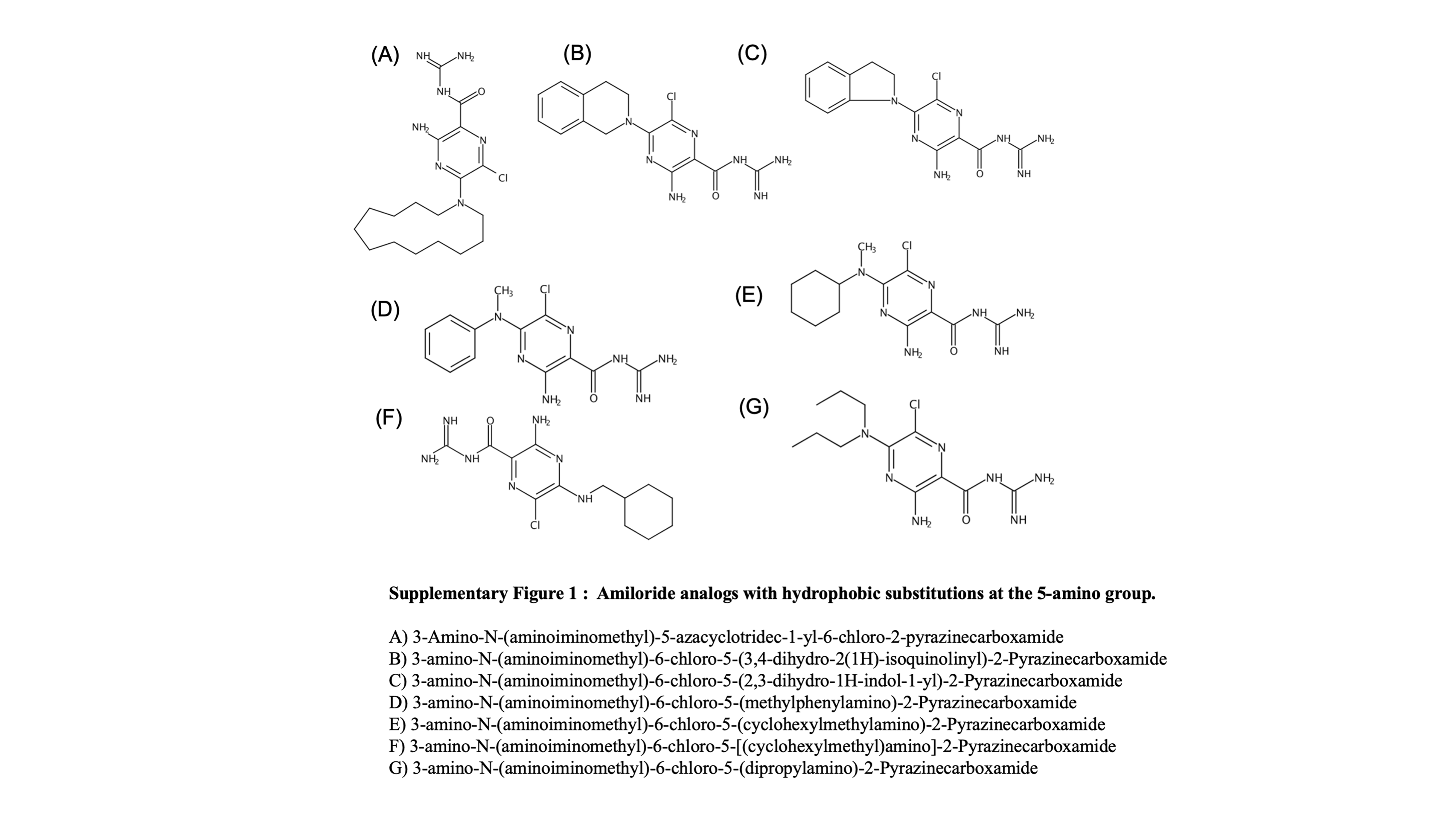

Supplement: Supplementary Figure 1 — Candidate amiloride analogs with hydrophobic substitutions at the 5-amino group of the pyrazine ring. (A) 3-Amino-N-(aminoiminomethyl)-5-azacyclotridec-1-yl-6-chloro-2-pyrazinecarboxamide; (B) 3-amino-N-(aminoiminomethyl)-6-chloro-5-(3,4-dihydro-2(1H)-isoquinolinyl)-2Pyrazinecarboxamide; (C) 3-amino-N-(aminoiminomethyl)-6-chloro-5-(2,3-dihydro-1H-indol-1-yl)-2-Pyrazinecarboxamide; (D) 3-amino-N-(aminoiminomethyl)-6-chloro-5-(methylphenylamino)-2-Pyrazinecarboxamide; (E) 3-amino-N-(aminoiminomethyl)-6-chloro-5-(cyclohexylmethylamino)-2-Pyrazinecarboxamide; (F) 3-amino-N-(aminoiminomethyl)-6-chloro-5-[(cyclohexylmethyl)amino]-2-Pyrazinecarboxamide; (G) 3-amino-N-(aminoiminomethyl)-6-chloro-5-(dipropylamino)-2- Pyrazinecarboxamide. [file Image_1.TIF]

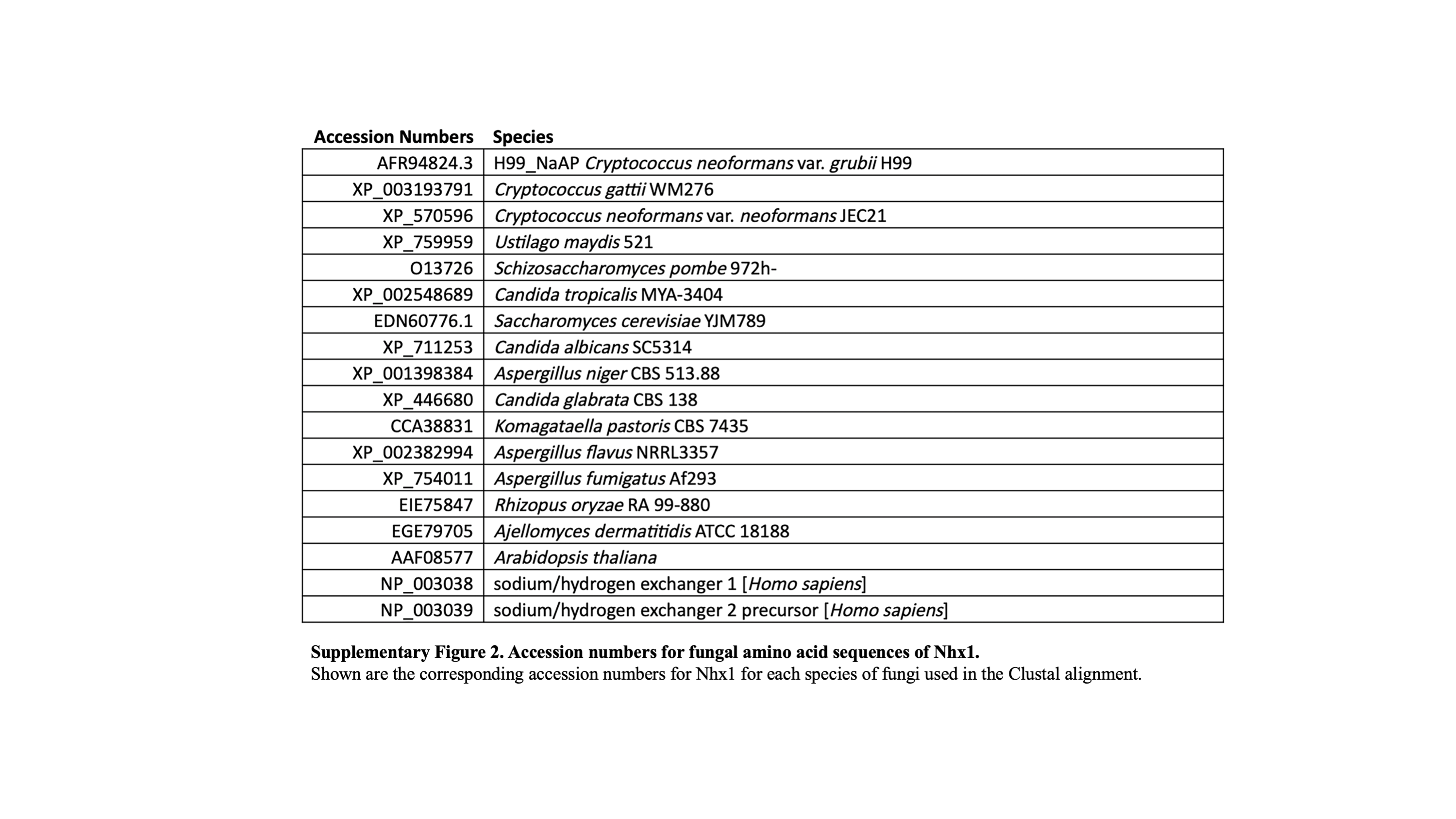

Supplement: Supplementary Figure 2 — Accession numbers for fungal amino acid sequences of Nhx1. Shown are the corresponding accession numbers for Nhx1 for each species of fungi used in the Clustal alignment. [file Image_2.TIFF]
